# Mother-to-child transmission of HIV in Kenya: A cross-sectional analysis of the national database over nine years

**DOI:** 10.1371/journal.pone.0183860

**Published:** 2017-08-29

**Authors:** Matilu Mwau, Priska Bwana, Lucy Kithinji, Francis Ogollah, Samuel Ochieng, Catherine Akinyi, Maureen Adhiambo, Fred Ogumbo, Martin Sirengo, Caroline Boeke

**Affiliations:** 1 Centre for Infectious and Parasitic Diseases Control Research, Kenya Medical Research Institute, Busia, Kenya; 2 Centre for Virus Research, Kenya Medical Research Institute, Nairobi, Kenya; 3 National AIDS and STIs Control Program, Ministry of Health, Nairobi, Kenya; 4 Clinton Health Access Initiative, Boston, Massachusetts, United States of America; University of New South Wales, AUSTRALIA

## Abstract

**Objective:**

To describe factors associated with mother-to-child HIV transmission (MTCT) in Kenya and identify opportunities to increase testing/care coverage.

**Design:**

Cross-sectional analysis of national early infant diagnosis (EID) database.

**Methods:**

365,841 Kenyan infants were tested for HIV from January 2007-July 2015 and results, demographics, and treatment information were entered into a national database. HIV risk factors were assessed using multivariable logistic regression.

**Results:**

11.1% of infants tested HIV positive in 2007–2010 and 6.9% in 2014–2015. Greater odds of infection were observed in females (OR: 1.08; 95% CI:1.05–1.11), older children (18–24 months vs. 6 weeks-2 months: 4.26; 95% CI:3.87–4.69), infants whose mothers received no PMTCT intervention (vs. HAART OR: 1.92; 95% CI:1.79–2.06), infants receiving no prophylaxis (vs. nevirapine for 6 weeks OR: 2.76; 95% CI:2.51–3.05), and infants mixed breastfed (vs. exclusive breastfeeding OR: 1.39; 95% CI:1.30–1.49). In 2014–2015, 9.1% of infants had mothers who were not on treatment during pregnancy, 9.8% were not on prophylaxis, and 7.0% were mixed breastfed. Infants exposed to all three risky practices had a seven-fold higher odds of HIV infection compared to those exposed to recommended practices. The highest yield of HIV-positive infants were found through targeted testing of symptomatic infants in pediatric/outpatient departments (>15%); still, most infected infants were identified through PMTCT programs.

**Conclusion:**

Despite impressive gains in Kenya’s PMTCT program, some HIV-infected infants present late and are not benefitting from PMTCT best practices. Efforts to identify these early and enforce evidence-based practice for PMTCT should be scaled up. Infant testing should be expanded in pediatric/outpatient departments, given high yields in these portals.

## Introduction

Without antiretroviral therapy (ART), approximately 50% of HIV-infected infants die by the age of two[1]. However, the use of elective caesarian section, the provision of highly active antiretroviral therapy (HAART) during pregnancy, and the avoidance of breastfeeding have reduced the HIV transmission rate to less than 2% and resulted in the near elimination of mother to child transmission (MTCT) [2, 3]. Unfortunately, barriers to healthcare and sub-optimal infant feeding and care practices still exist in resource-limited settings and continue to propagate transmission of HIV from mothers to infants. Identifying infected infants early and initiating them on ART as soon as possible after diagnosis is essential to slow the progression from HIV infection to AIDS and prolong the life of the patient[1].

Despite substantial progress in efforts to reduce MTCT of HIV in Kenya, HIV transmission through the end of breastfeeding remains at 17%, with approximately 13,000 new HIV infections among children in 2014[4]. Option B+, an approach to prevent MTCT of HIV by offering all HIV-positive pregnant women treatment for life and ART prophylaxis to all exposed infants, free of charge, has been scaled up through a phased rollout in Kenya starting in 2014; by October 2015 more than 90% of all sites with programs on prevention to mother-to-child transmission of HIV (PMTCT) or maternal, newborn, and child health had adopted it[5]. UNAIDS estimates suggest that greater than 90% of all pregnant women attending ANC in Kenya are tested for HIV[6]. However, gaps remain. Nearly half (42%) of women do not fully access antenatal care (at least 4 visits during pregnancy)[7], and 39% deliver outside of healthcare facilities[8]. Access to care is poorest in rural areas. Due in large part to this gap, an estimated 33% of HIV-infected pregnant women are not on treatment and early infant diagnosis of HIV (EID) testing coverage by two months of age in exposed infants was 72% in 2014[4]. Better understanding of these gaps and the remaining risk factors for HIV infection among infants who get tested is essential to eliminate MTCT in Kenya and neighboring countries.

This study used nearly a decade of nationwide data from a national HIV laboratory database of over 370,000 EID samples to assess the risk factors for HIV transmission in infants and to identify important predictors of HIV infection over time. We also assessed which facility entry points had the greatest yield for EID case detection of HIV-infected infants to inform resource allocation for HIV testing. While as a registry-based study, the data have some limitations, this is amongst the first studies of this scale examining risk factors for HIV infection in infants through the period of scale-up of Option B+ in sub-Saharan Africa.

## Materials and methods

### Study setting and design

The EID program in Kenya is overseen by the Ministry of Health. As of 2007, there was targeted testing of HIV-exposed symptomatic infants; in 2008–2009, as more resources became available for testing, the guidelines changed to test all HIV-exposed infants. The infant HIV testing algorithm as of 2012 in Kenya was as follows: a maternal or infant HIV antibody test was conducted at first visit for all children of unknown HIV status aged <18 months to establish HIV exposure status. If positive, an EID test was recommended. If the EID test was positive, the infant was started on ART and if negative, the infant received an HIV antibody test at 9 months (or earlier if child developed symptoms suggestive of HIV). If the antibody test was positive at 9 months, the infant received a confirmatory EID test. If the EID test was negative, the HIV antibody test was repeated at 18 months or 6 weeks after cessation of breastfeeding in a child >18 months[9]. EID testing coverage by 2 months of age in HIV-exposed infants in Kenya was estimated to be below 50% in 2011 and increased to 72% in 2014[4]. In 2015, the algorithm was updated to recommend an EID test at 6 weeks or first contact after 6 weeks[10]. Seven laboratories form the testing network and laboratory request forms are compiled into a national database across the laboratories. Thus, the database covers nearly all infants receiving EID testing in the country. Data from the database can be viewed on a national dashboard (http://eid.nascop.org/). Mean turnaround time from sample collection to results dispatch from the laboratory has changed over time but was 17 days as of July 2015[11]. This cross-sectional analysis was based on a retrospective review of all data stored in the national HIV database between January 2007 and July 2015.

### Clinical and laboratory procedures

Dried Blood Spots (DBS) were collected as part of routine care for infants with suspected or known HIV exposure as previously described[12]. Briefly, samples were collected under sterile conditions from infants using either a heel prick or finger prick depending on the age and weight of the infant. Sample collection occurred at several entry points including maternal and child health (MCH)/PMTCT clinics, the comprehensive clinic care (CCC)/patient support center (PSC), the outpatient department (OPD), and the pediatric ward. DBS filter papers were labeled and dried separately on a drying rack overnight. They were then packaged using glycine envelopes and sealed plastic bags under sterile conditions and sent to the testing laboratory by a courier system accompanied by a laboratory request form.

CobasAmpliPrep/CobasTaqMan_HIV-1 Qual or Abbot real-time HIV automated PCR test procedures were conducted on each sample based on the manufacturer’s guidelines, as described previously[12–14]. The CobasAmpliPrep/CobasTaqMan_HIV-1 Qual uses one dried blood spot (70uL) while the Abbot real-time HIV automated PCR testing uses two spots (140uL) of blood for the test. All positive samples were retested to confirm their status while a request for recollection of a new sample was made in cases where the test failed or number of blood spots was not sufficient to allow repeat confirmatory testing for positive samples.

Lab results were stored in an online accessible Laboratory Information System database provided by the Ministry of Health, along with the laboratory request form indicating the number of samples sent to the testing laboratory, the age and sex of the infant, mother’s HIV status, breastfeeding status, point of entry, medication given to the mother, and prophylaxis given to the baby. The database was retrospectively accessed to extract the relevant data and run these analyses.

### Study population

A total of 370,196 samples were successfully collected from infants visiting health facilities across all regions in Kenya between January 2007 and July 2015 and tested in seven national laboratories. 362 samples had the same patient ID number, came from the same facility, were tested on the same date, and were from an individual of the same age and sex as another sample; these were considered to be duplicate tests from the same infant and were excluded from the dataset. Due to improper DBS sample collection, packaging or labeling, 3228 samples were rejected and were therefore excluded from the analysis. Patients with age listed as a negative value or greater than or equal to 2 years (n = 765) were excluded from the main analysis; a secondary analysis was conducted setting age to missing in these individuals. Patients with missing data on other predictor variables were included in a missing indicator category. 365,841 patients had a valid test result and were included in the tables and primary analyses.

### Statistical analysis

Demographic data were summarized with descriptive statistics. The primary outcome was infant HIV status (infected vs. uninfected). We examined predictors of infant HIV status, including breastfeeding, age, prophylaxis, antiretroviral therapy and portal of entry. As a preliminary statistical analysis, continuous predictors were compared in relation to infant HIV result using Student’s T-tests if normally distributed or Mann-Whitney U tests if skewed. Categorical data were compared using the Fisher’s exact test or the chi square test.

Logistic regression was used to calculate univariate odds ratios of potential predictors in relation to infant HIV result and 95% confidence intervals. Variables that were significantly associated with infant HIV infection in univariate models were included in a multivariate logistic regression model. Finally, we combined all behavioral risk factors identified for infant HIV infection into one variable and assessed the increased odds of infection in an infant with multiple risk factors. The models accounted for clustering by health facilities using the vce(cluster) function for statistical analysis. Reference groups were selected based on the most common group and/or the standard of care in Kenya. The interaction between age and sex was examined and a Wald p-value for interaction was calculated. Separate logistic regression models were run by time period (2007–2010, prior to any Option B+ programs and when EID testing was limited and targeted; 2011–2013, when EID testing was scaled up and Option B+ was piloted at some sites; and 2014–2015, when Option B+ was rolled out nationally and EID coverage continued to expand) to assess differences over time as Kenya’s national testing and treatment program grew and developed. Significance levels were set at p<0.05. All analysis in this study was conducted using Stata Version 13 (StataCorp LP, College Station, Texas, USA).

### Quality assurance

Kenya has a national quality assurance program for the seven molecular laboratories. The program supports external quality assurance, training/registration of users, and harmonisation of operating procedures. All seven labs are enrolled and participate in the CDC Atlanta proficiency testing program. They also participate in a quarterly inter-laboratory EQA programme.

### Ethical approval

This study was approved by the Scientific Steering Committee and Ethical Review Committee of the Kenya Medical Research Institute (Protocol SSC No. 1066). The need for participant consent was waived. No identifiable patient information was included in the database, but facility-specific patient identification numbers were included. NASCOP considers the EID service as a national HIV program and a standard of care.

## Results

Patient characteristics and univariate and multivariate logistic regression analyses are presented in Table 1. Of the 365,841 infants in the dataset, 8.9% (32,441) were found to be HIV-infected. 50.4% of samples (of those with known sex) were from females. Data on sex was unavailable for 10.9% of samples. The median age at testing was 1.8 months (IQR: 1.5–5.0 months). Age at testing did not differ substantially between the sexes. 51.5% mothers were confirmed to be on highly active antiretroviral therapy (HAART; out of those with data on treatment regimen) and an additional 4.7% on interrupted HAART. 18.0% were on no treatment. 11.8% of infants were not on any HIV prophylaxis (among those with known prophylaxis status). Exclusive breastfeeding (EBF) was provided to 64.0% of infants (out of those with breastfeeding information), while 14.5% of infants were not breastfed. Mixed breastfeeding was practiced by 9.1% of infants, although only 60.4% of those infants were under 6 months of age and it is unknown whether the older children were previously exclusively breastfed and currently complementary feeding or were previously mixed breastfed. In addition, mixed breastfeeding decreased over time. 12.5% of infants were breastfed but did not have information on whether it was exclusive or mixed (hereafter referred to as “unspecified breastfeeding”). Approximately 72.3% of samples were collected via PMTCT/maternal and child health (MCH) clinic samples, while 18.8% were collected from CCC/ PSC, 3.5% from OPD, 1.2% from the pediatric ward and 0.9% from the maternity ward. The vast majority of samples across entry points were from patients ages 1 to 2 months, although the entry points with the most extreme peak in that age range were from PMTCT/MCH and CCC/PSC.

**Table 1:** Predictive factors for HIV positivity in 365,841 infants testing for HIV in Kenya.

Factor				Univariate		Multivariate	
		N	% HIV+	OR	95%CI	OR	95%CI
Gender	Male	161,682	8.4%	Ref		Ref	
	Female	164,259	8.9%	1.06	1.04–1.09	1.08	1.05–1.11
	Missing	39,900	10.4%	1.26	1.19–1.32	1.11	1.03–1.20
Age group	0–6 weeks	47,968	10.1%	2.43	2.25–2.63	1.72	1.59–1.86
	6 weeks-2 months	138,494	4.4%	Ref		Ref	
	2–6 months	86,877	9.4%	2.25	2.12–2.39	1.76	1.68–1.86
	6–9 months	35,888	11.9%	2.93	2.73–3.13	2.08	1.95–2.22
	9–18 months	46,834	16.3%	4.23	3.96–4.51	3.05	2.89–3.21
	18–24 months	4,599	21.6%	5.98	5.36–6.67	4.26	3.87–4.69
	Missing	5,181	8.3%	1.96	1.73–2.22	1.47	1.26–1.72
PMTCT Intervention	AZT from 14 weeks of pregnancy or later; AZT+3TC+sdNVP during labor; AZT+3TC for 7 days postpartum	41,394	6.5%	1.13	1.06–1.21	1.13	1.06–1.20
	HAART	125,166	5.8%	Ref		Ref	
	Interrupted HAART (HAART until end of breastfeeding)	11,437	7.0%	1.22	1.09–1.37	1.20	1.08–1.33
	None	43,837	15.2%	2.90	2.67–3.16	1.92	1.79–2.06
	Other	12,711	8.6%	1.53	1.37–1.72	1.23	1.10–1.36
	Single dose NVP Only	8,633	9.1%	1.62	1.45–1.80	1.32	1.20–1.45
	Missing	122,663	10.7%	1.94	1.80–2.10	1.53	1.43–1.63
Infant Prophylaxis	AZT+3TC for 7 days only	128	2.3%	0.57	0.17–1.90	0.40	0.12–1.35
	NVP during breastfeeding	50,505	6.3%	1.58	1.45–1.73	1.30	1.20–1.41
	NVP for 6 weeks (Mother on HAART or not breastfeeding)	67,429	4.0%	Ref		Ref	
	None	19,889	21.7%	6.56	5.83–7.39	2.76	2.51–3.05
	Other	5,689	8.4%	2.18	1.82–2.60	1.46	1.25–1.70
	Single dose NVP+AZT+3TC	3,436	8.1%	2.09	1.74–2.50	1.45	1.22–1.73
	Single dose NVP Only	21,541	5.9%	1.49	1.34–1.65	1.19	1.09–1.30
	Missing	197,224	10.2%	2.71	2.48–2.95	1.56	1.43–1.70
Breastfeeding	Exclusive breastfeeding	167,166	6.2%	Ref		Ref	
	Mixed breastfeeding	23,682	15.2%	2.73	2.48–3.00	1.39	1.30–1.49
	Breastfeeding, unspecified	32,609	11.3%	1.94	1.81–2.08	1.13	1.05–1.20
	No breastfeeding	37,856	9.7%	1.63	1.49–1.80	0.95	0.88–1.02
	Missing	104,528	10.6%	1.81	1.70–1.92	0.98	0.92–1.04
Entry point	Comprehensive clinic care/patient support center	54,257	9.1%	1.27	1.17–1.38	1.10	1.03–1.17
	Maternal and child health/PMTCT	208,834	7.3%	Ref		Ref	
	Maternity	2,505	9.0%	1.26	1.05–1.51	1.12	0.94–1.33
	Outpatient department	10,216	18.7%	2.93	2.68–3.19	1.65	1.52–1.79
	Other	9,753	12.6%	1.83	1.64–2.05	1.15	1.03–1.28
	Pediatric Ward	3,381	32.9%	6.23	5.26–7.39	3.32	2.82–3.92
	Missing	76,895	10.2%	1.44	1.34–1.55	0.92	0.87–0.99
Test year	2007	1,548	31.5%	6.61	5.90–7.41	3.72	2.85–4.85
	2008	18,257	10.1%	1.62	1.52–1.72	1.00	0.89–1.13
	2009	19,116	11.7%	1.91	1.81–2.03	1.37	1.21–1.54
	2010	57,584	10.7%	1.72	1.65–1.81	1.12	1.03–1.22
	2011	58,213	10.0%	1.61	1.53–1.69	1.08	0.99–1.18
	2012	55,063	8.2%	1.29	1.23–1.36	0.91	0.86–0.97
	2013	55,070	8.0%	1.25	1.19–1.31	0.99	0.94–1.06
	2014	58,495	7.1%	1.10	1.05–1.16	0.96	0.91–1.02
	2015	42,495	6.5%	Ref		Ref	

Univariate and multivariable logistic regression accounting for clustering by health facility. The multivariable model was adjusted for all factors in this table. The missing indicator method was used to account for missing data. Abbreviations: AZT: zidovudine. 3TC: lamivudine. Sd: single dose. NVP: nevirapine. HAART: highly active antiretroviral therapy. PMTCT: prevention of mother-to-child transmission of HIV.

In multivariate analyses, the odds ratio for HIV infection in females was 1.08 (95% CI 1.05–1.11) compared to males. When examining the sex differences in infection by age, there was a statistically significant difference between positivity in females and males through 6 months but no statistically significant difference in older children (p-value for interaction = 0.01). 10.1% of newborns less than 6 weeks of age were found to be infected with HIV. Compared to infants being tested at 6 weeks to 2 months (the majority of infants), the odds ratio of testing positive for HIV infection was 1.72 (95% CI: 1.59–1.86) in infants aged 0 to 6 weeks. However, most of those infants were listed as testing at 0 months, which may have represented a data entry error; sensitivity analyses in which these infants were considered to be missing age showed an attenuated association in this age group [OR changed to 1.28 (95% CI: 1.18–1.40)]. The percentage testing positive for infection was the lowest at 6 weeks to 2 months when the largest number of infants were tested, and then increased with age to 21.6% at 18 months to 2 years (76.3% of infants in this category were 18.0–18.5 months old). Compared to infants being tested at 6 weeks to 2 months, the odds ratio of testing positive for HIV infection was 1.76 (95% CI: 1.68–1.86) in infants 2 to 6 months, increasing to 4.26 (95% CI: 3.87–4.69) in infants 18–24 months. Infants whose mothers were not taking ART had nearly double the odds of the HIV infection when compared to those whose mothers were on treatment (multivariable-adjusted OR 1.92; 95% CI 1.79–2.06). For infants not on any prophylaxis, the odds ratio for HIV infection was 2.76 (95% CI 2.51–3.05) when compared to those who were on nevirapine (NVP) for six weeks whose mothers were on ART or were not breastfeeding. The percentage of infants on nevirapine for 6 weeks that tested positive was 4.0% vs. 21.7% of infants on no prophylaxis. Among infants whose mothers were on HAART, the differences in HIV infection by infant prophylaxis regimen were smaller but still substantial; 3.3% for those on nevirapine for 6 weeks and mother breastfeeding while 11.8% for those on no prophylaxis. Infants who were categorized as mixed breastfed had 1.39 (95% CI: 1.30–1.49) times the odds of HIV infection when compared to exclusively breastfed infants. There was not a statistically significant difference in HIV infection between exclusively breastfeeding and not breastfeeding. The highest yield for detecting infected patients was observed in the pediatric ward and the outpatient department. For infants who sought health services at the pediatric ward, the odds ratio of HIV infection was 3.32 (95% CI 2.82–3.92), while for those seen at the outpatient departments the odds ratio was 1.65 (95% CI 1.52–1.79) when compared to the MCH-PMTCT. However, given the difference in the volume of patients tested in each entry point, only 3.4% (5.3% in 2014–2015) of the HIV-infected infants were identified via the pediatric ward and 5.9% (9.0% in 2014–2015) through the outpatient department, whereas 46.9% (55.8% in 2014–2015) of patients were identified through MCH/PMTCT.

In a model combining sub-optimal behaviors for HIV transmission, the adjusted odds ratio of HIV infection for infants who did not receive infant prophylaxis, were mixed fed, and whose mothers did not receive any form of antiretroviral therapy (N = 2,550) was 7.10 (95%CI 5.64–8.93) compared to infants who received nevirapine for 6 weeks, were not breastfed, and whose mothers were on HAART (N = 3,325), and the odds were similar when compared to infants who received nevirapine, were exclusively breastfed, and mothers were on HAART [OR: 6.91 (95% CI: 6.14–7.78); S1 Table].

Table 2 shows multivariable-adjusted model results stratified by early and recent time period. The percentage of infants receiving an EID test who tested positive for HIV infection decreased over time; in 2007–2010, positivity was 11.1% while in 2014–2015, positivity was 6.9%. Most associations were consistent over time, although some associations varied in their strength.

**Table 2:** Risk factors for HIV positivity in 2007–2010 (N = 96,505; 11.1% HIV-positive) vs. 2011–2013 (N = 168,346; 8.8% HIV-positive) vs. 2014–2015 (N = 100,990; 6.9% HIV-positive) among infants testing for HIV in Kenya.

Factor		2007–2010				2011–2013				2014–2015			
		N	% HIV+	OR	95%CI	N	% HIV+	OR	95%CI	N	% HIV+	OR	95%CI
Gender	Male	37,305	10.8%	Ref		77,365	8.4%	Ref		47,012	6.7%	Ref	
	Female	37,639	11.4%	1.07	1.03–1.12	78,483	9.0%	1.09	1.05–1.13	48,137	6.9%	1.08	1.02–1.14
Age group	0–6 weeks	23,035	11.2%	1.50	1.31–1.71	17,115	9.9%	1.76	1.54–2.01	7,818	7.2%	1.79	1.59–2.03
	6 weeks-2 months	23,039	6.8%	Ref		63,735	4.6%	Ref		51,720	3.1%	Ref	
	2–6 months	24,858	11.1%	1.57	1.42–1.72	39,968	9.2%	1.71	1.60–1.82	22,051	7.9%	2.05	1.88–2.23
	6–9 months	11,959	13.6%	2.02	1.82–2.24	17,094	11.1%	1.89	1.75–2.04	6,835	11.0%	2.21	1.98–2.46
	9–18 months	11,455	16.6%	2.55	2.25–2.89	25,273	15.3%	2.76	2.58–2.96	10,106	18.5%	4.05	3.64–4.50
	18–24 months	982	20.6%	3.37	2.70–4.20	2,632	19.3%	3.60	3.17–4.08	985	28.7%	7.02	5.81–8.49
PMTCT Intervention	AZT from 14 weeks of pregnancy or later; AZT+3TC+sdNVP during labor; AZT+3TC for 7 days postpartum	8,559	8.7%	1.06	0.95–1.18	23,819	6.0%	1.15	1.05–1.26	9,016	5.8%	1.22	1.07–1.39
	HAART	21,979	9.1%	Ref		49,017	5.9%	Ref		54,170	4.4%	Ref	
	Interrupted HAART (HAART until end of breastfeeding)	805	10.2%	0.90	0.71–1.14	6,691	6.9%	1.24	1.09–1.41	3,941	6.5%	1.25	1.06–1.48
	None	15,254	12.0%	1.44	1.29–1.61	21,448	14.8%	2.06	1.86–2.28	7,135	23.3%	2.40	2.16–2.67
	Other	1,042	13.0%	1.24	1.01–1.52	8,428	8.2%	1.19	1.04–1.37	3,241	8.4%	1.46	1.25–1.71
	Single dose NVP Only	3,100	10.1%	1.22	1.04–1.43	4,307	9.0%	1.50	1.31–1.71	1,226	6.9%	1.20	0.93–1.56
Infant Prophylaxis	AZT+3TC for 7 days only	0		-	-	25	0.0%	-	-	103	2.9%	0.47	0.13–1.68
	NVP during breastfeeding	20	5.0%	0.59	0.03–10.16	28,385	6.5%	1.26	1.14–1.39	22,100	6.0%	1.26	1.13–1.40
	NVP for 6 weeks (Mother on HAART or not breastfeeding)	13	7.7%	Ref		27,478	4.4%	Ref		39,938	3.8%	Ref	
	None	35	11.4%	1.23	0.16–9.34	11,731	20.8%	2.57	2.29–2.89	8,123	23.0%	2.30	2.04–2.58
	Other	6	16.7%	1.52	0.08–30.12	2,567	10.4%	1.51	1.22–1.87	3,116	6.8%	1.28	1.04–1.57
	Single dose NVP+AZT+3TC	36	0.0%	-	-	2,349	6.8%	1.19	0.97–1.45	1,051	11.2%	2.05	1.55–2.71
	Single dose NVP Only	19	0.0%	-	-	13,256	6.4%	1.23	1.10–1.37	8,266	5.0%	1.05	0.92–1.20
Breastfeeding	Exclusive breastfeeding	13,199	12.4%	Ref		83,080	6.5%	Ref		70,887	4.7%	Ref	
	Mixed breastfeeding	5,112	9.7%	0.74	0.65–0.83	12,330	14.6%	1.39	1.27–1.52	6,240	21.1%	1.84	1.67–2.03
	Breastfeeding, unspecified	18,491	10.3%	0.79	0.71–0.89	9,832	13.0%	1.25	1.13–1.37	4,286	12.2%	1.17	1.03–1.33
	No breastfeeding	11,134	9.4%	0.66	0.59–0.74	19,213	9.9%	1.02	0.93–1.12	7,509	9.8%	1.03	0.88–1.20
Entry point	Comprehensive clinic care/patient support center	11,539	12.7%	1.22	1.12–1.32	26,043	8.9%	1.03	0.94–1.13	16,675	6.9%	1.01	0.91–1.11
	Maternal and child health/PMTCT	45,312	9.8%	Ref		93,876	7.4%	Ref		69,646	5.5%	Ref	
	Maternity	534	11.4%	1.19	0.82–1.72	1,191	9.2%	1.07	0.86–1.33	780	7.2%	0.96	0.71–1.30
	Outpatient department	1,210	15.4%	1.44	1.18–1.75	6,537	16.9%	1.55	1.38–1.73	2,469	25.2%	1.81	1.60–2.04
	Other	4,444	11.3%	1.09	0.96–1.24	3,753	14.8%	1.40	1.19–1.66	1,556	10.7%	0.79	0.58–1.08
	Pediatric Ward	1,128	26.1%	2.63	2.09–3.32	1,337	33.7%	3.53	2.86–4.35	916	40.1%	3.90	3.08–4.93

Multivariable logistic regression accounting for clustering by health facility. A separate model was run for each time period. The multivariable model was adjusted for all factors in this table plus test year. The missing indicator method was used to account for missing data; to save space, missing categories are not shown. Abbreviations: AZT: zidovudine. 3TC: lamivudine. Sd: single dose. NVP: nevirapine. HAART: highly active antiretroviral therapy. PMTCT: prevention of mother-to-child transmission of HIV

The proportion of mothers not on ART during pregnancy (among those with treatment data) decreased dramatically over time, from 30.1% in 2007–2010 to 9.1% in 2014–2015. The percentage of infants born to mothers on HAART who were HIV-infected decreased from 9.1% in 2007–2010 to 4.4% in 2014–2015. Infant prophylaxis was not widely available in 2007–2010 so data was not routinely collected, but between 2011–2013 and 2014–2015 the proportion of infants receiving no prophylaxis decreased from 13.7% to 9.8%.

The higher prevalence of HIV infection among older children compared to younger children was stronger after scale-up of Option B+ (adjusted OR comparing children aged 18–24 months to children 6 weeks to 2 months in 2014–2015: 7.02; 95% CI: 5.81–8.49).

Mixed breastfeeding decreased from 10.7% of infants in 2007–2010 (plus 38.6% unspecified breastfeeding) to 7.0% (plus 4.8% unspecified breastfeeding) in 2014–2015. There may have been differences in the way that the breastfeeding categories were defined/reported over the years, as in 2007–2010”mixed breastfeeding” appeared to be *protective* compared to exclusive breastfeeding whereas in more recent years it was harmful. Not breastfeeding appeared protective compared to exclusive breastfeeding in 2007–2010 (OR: 0.66; 95% CI: 0.59–0.74) while there was no association in 2014–2015. Finally, associations between entry point and HIV have changed over time, particularly in that the pediatric ward in more recent years had an extremely high yield for identifying infected patients.

There were no substantial differences in the study findings when patients with age listed as a negative value or greater than or equal to 2 years were included in the analysis with age listed as missing.

## Discussion

For this study, we analysed a comprehensive national dataset from Kenya covering nearly a decade to describe the determinants of HIV status in infants below 2 years of age at testing. We observed greater odds of HIV infection in females, older children, infants whose mothers received no PMTCT intervention, infants receiving no HIV prophylaxis, and infants who were mixed breastfed. Exposure to risky practices (mother not on HAART, no infant prophylaxis, mixed breastfeeding) was associated with a seven-fold higher odds of HIV-positivity compared to exposure to recommended practices (mother on HAART, infant on nevirapine for six weeks, no breastfeeding). With PMTCT/EID program growth and more patients in care, there has been a decrease in risky practices over time, but even the recent data suggests that some infants are not reached in time for prevention; these findings would likely be more striking in other countries in sub-Saharan Africa where PMTCT programs are not as strong. Finally, the pediatric ward and outpatient department had the highest testing yield across entry portals, but most HIV-infected infants were identified through PMTCT/MCH programs.

Most importantly, many infants were not identified as infected until older ages and received sub-optimal prevention practices (lack of HAART for mother, infant prophylaxis, exclusive breastfeeding or exclusive replacement feeding). Option B+ has been scaled up in recent years and the number of new pediatric infections in Kenya has decreased by 29% from 2009 to 2014[4]. Yet hundreds to thousands of infants are still becoming infected each year because their mothers are not enrolled in care. Barriers to testing and care include distance to health facility and transportation costs, facility inefficiencies, such as stock-outs and long wait times, and persistent shamefulness and stigma[15]. Expanded testing efforts in adolescent and early adult women and increased HIV self-testing availability may help to expand case-finding in women of childbearing age.[16] Many pregnant women do not come to health facilities for antenatal care until late in pregnancy[17] if at all; a high proportion of women deliver outside of facilities. HIV-infected women who deliver outside of facilities tend to have lower income and be less educated and less likely to be on treatment[18], meaning that their infants are at especially high risk. Pilot interventions that have been shown to improve PMTCT program coverage, retention, and quality include mHealth tools[19] such as SMS[20], rapid results initiatives[21], systems engineering approaches[22], and efforts to reduce health provider absenteeism[23]; these could be considered in areas struggling with program performance. In addition, there likely remain inconsistencies with health provider care among women and infants enrolled in PMTCT programs. A case-control study in Western Kenya observed that Infants were more likely to be infected with HIV if their provider did not follow maternal and infant ART guidelines[24]. Ensuring reinforcement of guidelines through periodic re-trainings and supportive supervision, as well as strong supply chain management systems will be important to continue to strengthen the PMTCT program.

We observed HIV infections in some infants despite their mothers being on HAART; the percentage of infected infants in this category declined over time as drug regimens changed to 4.4% in 2014–2015. Possible reasons for transmission in these infants include late treatment initiation in mothers, treatment nonadherence, lower efficacy on certain treatments, drug resistance, and stock-outs. This database does not contain information on these factors and therefore we are only able to speculate on the relative contribution of each one. Some mothers had interrupted HAART, indicating that non-adherence to treatment may be another barrier to care and testing. Strong adherence support programs are also important to minimize transmission during pregnancy. Innovative methods including community adherence groups[25] and transport reimbursement for low-income patients[26] may help to improve drug adherence and retention in PMTCT programs. New treatment regimens for mothers and prophylaxis options for infants will likely influence transmission rates in the future.

Thousands of infants are still exposed to the sub-optimal practice of mixed breastfeeding each year in Kenya, and these infants had worse outcomes compared to exclusively breastfed infants and infants not given any breastmilk. The dangers posed by mixed feeding have been well-described in the literature[27–31]. Besides pointing to a gap in program coverage, these findings illustrate a need for enhanced education of the public that this practice is detrimental. The Kenyan National Demographic and Health Survey noted that by 2014 exclusive breastfeeding had increased to 61% in infants less than 6 months, a near doubling from 32% in 2009[32]. Yet a recent study among women in Nairobi found that fear of stigma and discrimination around HIV was a persistent barrier to exclusive breastfeeding[33]. In this study, authors concluded that health education and counseling as well as male partner support helped to mitigate these issues. Other studies have shown that intensive counseling is a viable way to increase EBF in resource-limited settings[34], although in an intervention study in Nairobi, breastfeeding counseling alone did not increase EBF[35]. Regardless, there is more work to be done to reduce mixed breastfeeding in infants younger than 6 months.

In this study we found that HIV positivity did not differ significantly between EBF and exclusive replacement feeding (ERF) after adjustment for other factors, even when adjusted for age. This was surprising, as exclusive replacement feeding should significantly reduce HIV transmission. It is possible that due to stigma, or the costs associated with ERF, some of those who said they provided replacement feeding for their infants also breastfed from time to time, leading to some measurement error in this category.

Infants who entered care and treatment through outpatient departments or the pediatric wards were more likely to be HIV-infected than those entering through any other service point. This finding is not surprising, as those infants come to hospital due to illnesses which may include, or could be related to, HIV, and supports the latest Kenyan guidelines stating that all children presenting for care should be offered an HIV test[36]. In addition, testing in these infants was likely offered in a targeted fashion; for example, symptomatic infants were more likely to be tested. The high proportion of infected patients suggests that these entry points may be important for expanded infant testing to identify more HIV-exposed infants that are not reached by routine testing through PMTCT programs. In the future, EID detection efforts could be expanded further to better cover patients in alternative entry points such as OPD and pediatric wards to identify those who are missed by PMTCT testing programs, given the high yield in these areas. This is in line with current WHO recommendations, which state that in settings with generalized epidemics, HIV testing should be performed on infants with unknown HIV status who are admitted to inpatient and nutrition wards and offered to infants with unknown HIV status at outpatient and immunization clinics[37]. Increased resources for testing at these portals may be important. However, it is important to note that the greatest numbers of cases are still detected through MCH/PMTCT programs, despite the lower yields in these portals, and these programs should continue this important work. Of note, a number of studies on improving Kenya’s EID program are recently completed or still ongoing[38–41]. Through continued national monitoring efforts in Kenya and scaling up such efforts in other countries, we can continue to work to minimize transmission of HIV to infants.

Overall, we observed slightly higher odds of positivity in female infants compared to male infants from birth to six weeks. While this small difference could be due to chance, this phenomenon has been described previously and there are theories that female infants may be more susceptible to HIV infection in the in-utero and peripartum period [42–44]. While the exact mechanism is not known, hypotheses include chromosomal affinity and a higher rate of male intrauterine death among HIV-infected fetuses, leading to females being more likely to be HIV infected at birth. In this database, females did not appear to be testing later than males, so age at testing did not seem to explain the imbalance. Confirmation in other studies and further investigation is needed to better understand this phenomenon.

This study has a number of limitations. Firstly, it is observational and cross-sectional; thus, causal relationships cannot be inferred. We did not have access to data on all predictors of infant HIV transmission and all potential confounders. In addition, the study data come from a national health registry system and were not collected for the purpose of a research study; thus, there were large amounts of missing data for some indicators and some inaccuracies are possible. Along those lines, given the vagueness of the patient identification numbers at some facilities, we were unable to assess tests from the same individuals or calculate a seroconversion rate among patients with multiple tests. Strengthening patient identification systems and data entry would further maximize the utility of this database. This analysis did not include outcome data on HIV-infected infants following diagnosis; linkage to care and retention in care for these infants is an important area for future research. Finally, the data is based on infants who were tested for HIV, which, while covering >70% of exposed infants, does not include the infants outside of care who are at highest risk of HIV transmission. This also means that comparisons between groups cannot be interpreted as nationally representative, do not necessarily represent transmission, and are in part a product of testing coverage. The strengths of this study include its large size, numerous risk factors examined, and comprehensiveness across Kenya over nearly a decade.

## Conclusions

This study emphasizes the importance of national health registry systems to monitor the success of programs such as Option B+ and to inform testing for EID. Given the relative success of Kenya’s national program, the associations are likely to be even more striking in other countries in sub-Saharan Africa with less successful PMTCT programs. We have confirmed risk factors previously seen on a smaller scale in this national dataset, and illustrated the impact of exposure to multiple risk factors overall and over time. The findings point to a need for resources to continue to reinforce best treatment and prophylaxis prescribing behavior, promote exclusive breastfeeding, and test infants early in life and at testing portals with greater case-finding. Routine point of care testing will be important to dramatically reduce time to treatment initiation for HIV-infected infants. Most importantly, efforts must continue to reach patients that are not coming for PMTCT programs.

## Supporting information

S1 Table Sub-optimal behaviors in relation to HIV positivity in 365,841 infants testing for HIV in Kenya. Univariate and multivariate logistic regression accounting for clustering by health facility. The multivariable model was adjusted for sex, age, entry point, and year. The missing indicator method was used to account for missing data. Abbreviations: NVP: nevirapine. HAART: highly active antiretroviral therapy. PMTCT: prevention of mother-to-child transmission of HIV.
(DOCX)
