# Supplementary material for: Mother-to-child transmission of HIV in Kenya: A cross-sectional analysis of the national database over nine years
Source: PLoS One. 2017 Aug 29;12(8):e0183860. doi: 10.1371/journal.pone.0183860 (PMC5574578; doi:10.1371/journal.pone.0183860)
Supplement: S1 Table — Univariate and multivariate logistic regression accounting for clustering by health facility. The multivariable model was adjusted for sex, age, entry point, and year. The missing indicator method was used to account for missing data. Abbreviations: NVP: nevirapine. HAART: highly active antiretroviral therapy. PMTCT: prevention of mother-to-child transmission of HIV. (DOCX) [file pone.0183860.s001.docx]

**Supplementary Table 1**. **Sub-optimal behaviors in relation to HIV positivity in 365,841 infants testing for HIV in Kenya.**

| **Behaviors** |  |  | **Univariate** | | **Multivariate** | |
| --- | --- | --- | --- | --- | --- | --- |
|  | N | % HIV+ | OR | 95%CI | OR | 95%CI |
| Mother on HAART, infant NVP for 6 weeks, **not** breastfed | 3,325 | 4.3% | Ref |  | Ref |  |
| No PMTCT intervention, no infant prophylaxis, mixed feeding | 2,550 | 35.0% | 12.08 | 9.47-15.40 | 7.10 | 5.64-8.93 |
| All others | 359,966 | 8.7% | 2.14 | 1.70-2.70 | 1.97 | 1.60-2.42 |
|  |  |  |  |  |  |  |
| Mother on HAART, infant NVP for 6 weeks, **exclusively** breastfed | 50,063 | 3.1% | Ref |  | Ref |  |
| No PMTCT intervention, no infant prophylaxis, mixed feeding | 2,550 | 35.0% | 16.60 | 14.85-18.57 | 6.91 | 6.14-7.78 |
| All others | 313,228 | 9.6% | 3.26 | 2.96-3.59 | 2.05 | 1.88-2.23 |

Univariate and multivariate logistic regression accounting for clustering by health facility. The multivariable model was adjusted for sex, age, entry point, and year. The missing indicator method was used to account for missing data. Abbreviations: NVP: nevirapine. HAART: highly active antiretroviral therapy. PMTCT: prevention of mother-to-child transmission of HIV.
